# Purification, Characterization and Antitumor Activities of a New Protein from *Syngnathus acus*, an Officinal Marine Fish

**DOI:** 10.3390/md10010035

**Published:** 2011-12-30

**Authors:** Mengyue Wang, Yuxiao Nie, Ying Peng, Fen He, Jingyu Yang, Chunfu Wu, Xiaobo Li

**Affiliations:** 1 School of Pharmacy, Shanghai Jiao Tong University, Shanghai 200240, China; Email: mywang@sjtu.edu.cn (M.W.); nieyuxiaolw@hotmail.com (Y.N.); ypeng@sjtu.edu.cn (Y.P.); fen_he@hotmail.com (F.H.); 2 School of Pharmacy, Shenyang Pharmaceutical University, Shenyang 110016, China; Email: yangjingyu2006@gmail.com (J.Y.); chunfuw@gmail.com (C.W.)

**Keywords:** *Syngnathus acus*, purification, characterization, antitumor, protein

## Abstract

Discovery and development of new antitumor agents from abundant marine fish are attracting an increasing interest. In the present study, we extracted and purified a novel antitumor protein Syngnathusin from the whole body of *Syngnathus acus* L., a precious marine fish traditionally used for tumors. Syngnathusin was comprised of 16 kinds of amino acids, mainly acidic amino acids. Its molecular weight was 67.3 kDa and its isoelectric point was 4.57. The *N*-terminal amino acid sequence of Syngnathusin was determined to be Lys-Arg-Asp-Leu-Gly-Phe-Val-Asp-Glu-Ile-Ser-Ala-His-Tyr and showed no significant homology with the known proteins. Syngnathusin could significantly inhibit the growth of A549 and CCRF-CEM cells. However, the obvious proliferation inhibition against human non-tumor cell lines was not observed. Flow cytometry, morphologic assessment and comet assay revealed that Syngnathusin could induce apoptosis in A549 and CCRF-CEM cells and strongly cooperated with MTX. Syngnathusin could inhibit the growth of S180 tumor transplanted in mice. Syngnathusin may be developed as a novel, selective and effective antineoplastic agent.

## Abbreviations

CTX: cytoxan
5-Fu: 5-fluorouracil
HCF: human uterine cervical canal fibroblast
HE-21: human embryonic fibroblast
HFL-1: human embryonic lung fibroblast
HL-7702: human normal liver cells
IEF-PAGE: isoelectric focusing-polyacrylamide gel electrophoresis
MALDI-TOF-MS: matrix-assisted laser desorption/ ionization time of flight mass spectrometry
MTT: methyl thiazolyl tetrozolium
MTX: methotrexate
PBMC: human normal peripheral blood mononuclear cells
PI: propidium iodide
PVDF: polyvinylidene difluoride
Rp-HPLC: reversed-phase high performance liquid chromatography
RPTEC: human normal renal proximal tubule epithelial cells
SDS-PAGE: sodium dodecylsulphate-polyacrylamide gel electrophoresis
SI: Synergism index
Tris: trisamine

## 1. Introduction

The modern research on traditional Chinese drugs has for a long time largely focused on the micromolecular constituents. However, recently, more and more researches have revealed some proteins and polypeptides e.g., lumbrukinase, hirudin and trichosanthin, possess the potent activities also. Some proteins and polypeptides from traditional Chinese drugs, especially from animal origin medical material, such as anthoplerintoxin, katsutoxin, snake venom, melittin, dolastatins and didemnins, exhibit significant antitumor activities [1,2]. Some experimental antitumor proteins derived from animal origin medical material have entered the clinical trials [3]. Marine fish, about 13,000 species, are a large and diverse group of animals containing high levels of protein, and more than 100 species are traditional medicines for tumors. However, only a tiny part of marine fish species were analyzed chemically and explored. Furthermore, most of the natural products derived from marine fish were evaluated and developed as functional food and these kinds of products were dominated by low molecular-weight compounds. Recently, a few proteins from marine fish such as shark, globefish, devil ray (*Manta birostris*), king crab, and hairy clam (*Arca subcrenata*) were reported to possess the potent antitumor activity [4,5,6]. Protamine and fish anti-bacterial peptides were also found to inhibit tumors [7,8]. Marine fish proteins represent an essentially unexploited resource for the discovery and development of potential antitumor drugs. 

In screening for antitumor protein from marine fish, pipefish have drawn much attention for their life characteristic and medical property. Pipefish, like their seahorse relatives, are remarkable for their adaptations for parental care, with females depositing eggs in male’s specialized incubating area where embryos are protected, nourished and osmoregulated. As an important medical pipefish with a medicinal history of more than 500 years, *Syngnathus acus* L. has been recorded in the Chinese Pharmacopoeia for cancer and impotency [9]. *S. acus*, widely distributed in the Pacific, Indian, Atlantic Ocean and Mediterranean Sea, contains a high level of protein, fatty acids and a small quantity of steroids [10,11]. The modern research found that the lipid-soluble constituents had the antitumor activity *in vitro* [12], harmonic effect [13,14] and anti-fatigue effect [15,16]. In traditional Chinese medicine, medical pipefish was used as aqueous extract for various cancers and other diseases. Some research also revealed that the water extract possessed the antitumor activity [17,18,19]. So, the water-soluble constituents in *S. acus*, especially proteins, deserve thorough investigation. However, the relevant research has not been reported yet. In the present study, antitumor protein in *S. acus* was investigated for the first time. As result, a novel protein Syngnathusin was purified and found to possess the potent antitumor *in vivo* and *in vitro*. 

## 2. Results and Discussion

### 2.1. Bioassay-Guided Isolation *in Vitro*

Crude protein of *Syngnathus acus* (CPSA) was isolated by ammonium sulfate saturation and then subjected to the antiproliferative assay against tumor cells. The results showed that CPAS suppressed the proliferation of A549, and CCRF-CEM cells, with the IC_50_ values less than 500 μg/mL (Table 1).

**Table 1:** The antiproliferative activities of protein samples against four tumor cell lines (IC_50_, μg/mL ± SD, *n* = 3).

Sample	Yield (mg) ^a^	Tumor Cell Lines			
		A549	L1210	CCRF-CEM	LoVo
CPAS	1289	232.5 ± 47.1	2369.8 ± 104.7	438.6 ± 130.7	3125.1 ± 432.0
P1	189	1433.6 ± 216.2	3077.1 ± 280.5	1257.7 ± 141.8	4561.4 ± 216.5
P2	266	897.4 ± 155.7	1564.3 ± 233.9	1021.6 ± 213.3	935.0 ± 122.7
P3	83	1561.0 ± 185.4	906.6 ± 214.0	1309.5 ± 176.4	3201.3 ± 240.1
P4	168	84.9 ± 17.7	1446.1 ± 153.7	215.3 ± 38.1	1706.4 ± 186.7
P5	56	1035.6 ± 147.9	2876.6 ± 178.4	710.5 ± 164.2	2543.3 ± 307.4
S1	110	371.4 ± 83.5	1489.7 ± 211.9	688.1 ± 235.5	2359.9 ± 187.2
S2	27	27.6 ± 5.3	416.3 ± 91.5	44.9 ± 6.2	814.7 ± 143.6

^a^ The weight of protein extracted from 100 g medical material.

**Figure 1:**
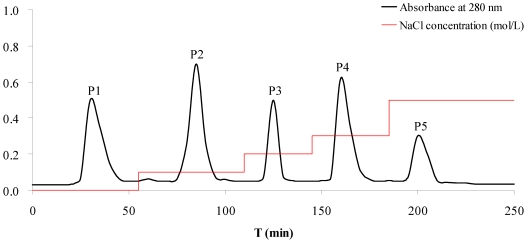
Purification of the crude protein of *S. acus* by ion-exchange chromatography on a DEAE-Sepharose Fast Flow column. Column dimensions: 2.5 cm × 50 cm. Flow rate: 1.0 mL/min. The elution profile was monitored by spectrophotometry at 280 nm. Peak P1, P2, P3, P4 and P5 were eluted by 0, 0.1, 0.2, 0.3 and 0.5 M NaCl prepared in 20 mM Tris-HCl buffer (pH 8.0), respectively.

In order to screen the antitumor components, CPAS was purified by ion-exchange chromatography. Five A280 nm peak fractions, P1, P2, P3, P4 and P5, were obtained through elution with increasing NaCl concentrations on DEAE Sepharose column (Figure 1). In the antiproliferative evaluation of five fractions above, the fraction P4 exhibited the significant inhibition on the proliferation of A549 and CCRF-CEM cells with the IC_50_ values of 84.9 and 215.3 μg/mL, respectively (Table 1). The other fractions did not obviously inhibit the tumor cells’ proliferation. The results suggested that the fraction P4 deserved the further purification in order to elucidate the active constituents in *S. acus*.

**Figure 2:**
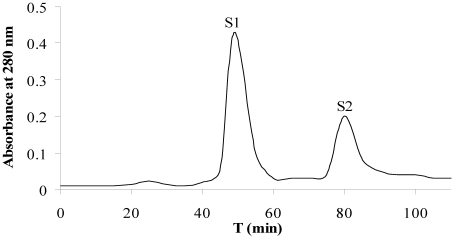
Purification of the DEAE chromatographic fraction P4 by gel filtration chromatography on a Superdex 75 column. Column dimensions: 2.0 cm × 30 cm. The elution was performed with 20 mM Tris-HCl containing 0.2 M NaCl at a flow of 1.0 mL/min. The elution profile was monitored by reading the absorbance at 280 nm.

Fraction P4 was subjected to gel filtration chromatography for the further purification. Two A280 nm peak fractions, S1 and S2, were obtained (Figure 2). The results of the antitumor activity evaluation *in vitro* suggested the fraction S2 had the potent activity to suppress the proliferation of tumor cells of A549 and CCRF-CEM, with the values of IC_50_ less than 50 μg/mL; while the fraction S1 only showed the weak inhibition proliferation activity (Table 1). 

In order to determine whether protein S1 and S2 exhibit the proliferation inhibition against human non-tumor cells, six non-tumor cell lines were treated by protein samples. The IC_50_ values were more than 10 mg/mL, indicating protein S1 and S2 had no obvious inhibition proliferation activity to human non-tumor cells (Table 2). For the higher antitumor activities *in vitro*, the further investigation of protein S2 is warranted. 

**Table 2:** The antiproliferative activities of protein samples against six human non-tumor cell lines (IC_50_, mg/mL ± SD, *n* = 3).

Sample	Non-Tumor Cell Lines					
	HCF	HE-21	HFL-1	HL-7702	PMBC	RPTEC
S1	30.1 ± 4.5	14.5 ± 3.1	25.3 ± 3.6	23.1 ± 4.3	28.9 ± 5.2	37.4 ± 6.2
S2	21.4 ± 2.3	33.9 ± 2.8	17.9 ± 1.8	26.3 ± 3.0	40.6 ± 5.3	32.5 ± 5.8

### 2.2. Characterization of Purified Protein

**Figure 3:**
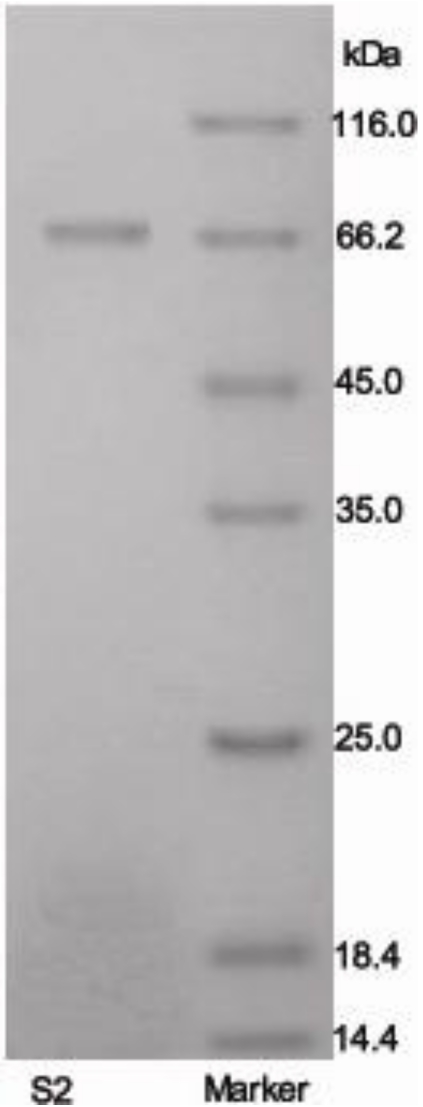
Purity and molecular mass analysis of protein S2 by SDS-PAGE using 5% stacking and 12% resolving polyacrylamide gel.

**Figure 4:**
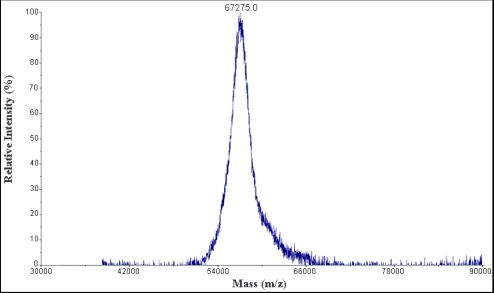
The MALDI-TOF-MS analysis of protein S2 (positive ion mode).

**Figure 5:**
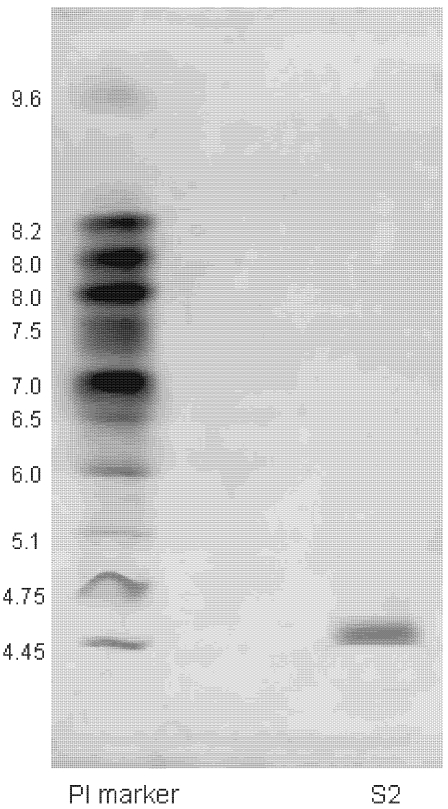
Isoelectric point determination of protein S2 by using immobilized pH gradient isoelectric focusing-polyacrylamide gel electrophoresis.

**Figure 6:**
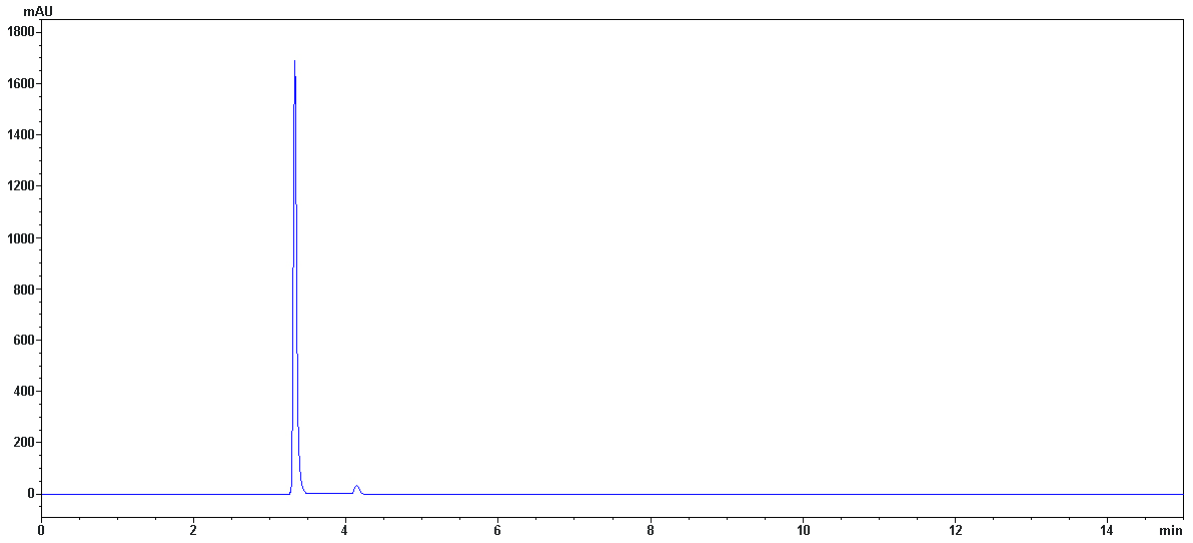
Rp-HPLC profile of S2. Performed on an Agilent 1200 HPLC system fitted with a C4 Hypersil column (5 μm, 300 Å, 4.6 mm × 250 mm). The elution solvent system was water-trifluoroacetic acid (solvent A; 100:0.005, v/v) and acetonitrile-trifluoroacetic acid (solvent B; 100:0.005, v/v). Isocratic elution 97% of solvent A for 15 min at flow rate of 1.0 mL/min. Detection wavelength was set at 280 nm and column temperature was 25 °C.

The molecular weight of protein S2 was estimated by SDS-PAGE, with molecular weight marker ranging from 14.4 to 116.0 kDa as standard. According to the calibration curve, the molecular weight of S2 was 67 kDa (Figure 3). The accurate molecular weight was 67.3 kDa determined by MALDI-TOF-MS (Figure 4). The IEF-PAGE result showed that the isoelectric point of protein S2 was 4.57 (Figure 5). Protein S2 exhibited a single band in both SDS-PAGE and IEF-PAGE, and showed a single peak in Rp-HPLC (Figure 6). The results indicated protein S2 was a homogeneous protein.

No glucose was detected in protein S2 by the carbohydrate content assay, indicating it was a nonglycoprotein. The amino acid analysis showed that protein S2 contained 16 kinds of amino acid, mostly acidic amino acid. Protein S2 was particularly rich in aspartic acid, glutamic acid, histidine and phenylalanine (Table 3). 

**Table 3:** The amino acid composition of protein S2.

Amino Acid	Weight (%)	Mol (%)
Ala	5.08	7.51
Arg	5.89	4.45
Asp	14.76	14.60
Cys	0.30	0.33
Glu	12.57	11.32
Gly	4.80	8.43
His	10.19	8.65
Ile	4.27	4.29
Leu	6.73	6.76
Lys	6.49	5.85
Met	0.79	0.70
Phe	10.59	8.44
Ser	4.54	5.68
Thr	3.23	3.57
Tyr	3.87	2.81
Val	5.89	6.62
Total	99.99	100.01

The *N*-terminal 14 amino acids sequence of S2 was determined to be Lys-Arg-Asp-Leu-Gly-Phe-Val-Asp-Glu-Ile-Ser-Ala-His-Tyr, indicating that its *N*-terminus was unblocked. This sequence was blasted in NCBI database. The results showed that it was a novel protein, since a data bank search could not detect any homologue with the available sequence. Protein S2 was named Syngnathusin, according to its origin.

### 2.3. Antitumor Activities

#### 2.3.1. Antitumor Activities *in Vivo*

**Figure 7:**
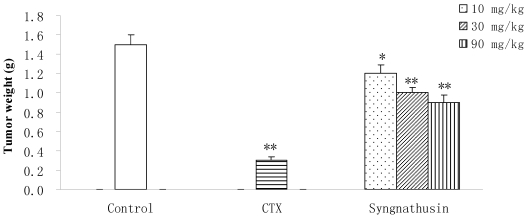
Effect of protein Syngnathusin on the tumor weight (*n* = 10). Compared with NS, * *p* ≤ 0.05, ** *p* ≤ 0.01.

Protein Syngnathusin inhibited the growth of implanted S180 solid tumors in mice at doses from 10 mg/kg to 90 mg/kg in a dose-dependent manner. Its antitumor activity *in vivo* was very obvious (inhibition ration surpassed 30%), indicating that the efficiency of Syngnathusin was more apparent *in vivo* studies than *in vitro* (Figure 7). However, the apparent antitumor activity of Syngnathusin was not observed in H22 mice cancer model (data not given), indicating the antineoplastic activtity of Syngnathusin was selective. 

#### 2.3.2. Induction of Apoptosis in Tumor Cells

##### 2.3.2.1. Effect on Tumor Cells Cycle

**Figure 8:**
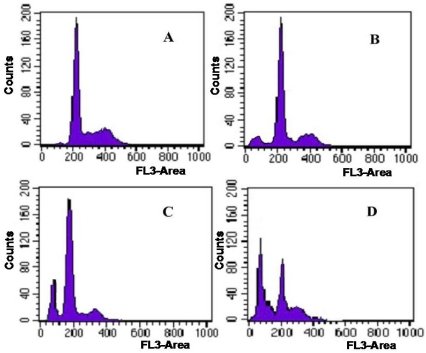
Effect of protein Syngnathusin on A549 cell cycles. (**A**) blank; (**B**) 0.04 mg/mL for 24 h; (**C**) 0.2 mg/mL for 24 h; (**D**) 1.0 mg/m for 24 h.

To further evaluate the antitumor activity of Syngnathusin, the effect on the cell cycle phase distribution of A549 cells in the cell cycle was examined by flow cytometry analysis. The percentage of apoptotic cells was performed by PI staining. Compared with the control group (0.7%), the proportion of apoptotic cells in sample treated group increased progressively in A549 cells. When the concentration arrived at 200 and 1000 mg/mL, the proportion of apoptotic cells was 20.4 and 43.4%, respectively (Figure 8). The result showed that Syngnathusin could induce the apoptosis in a dose-dependent manner.

##### 2.3.2.2. Comet Assay

**Figure 9:**
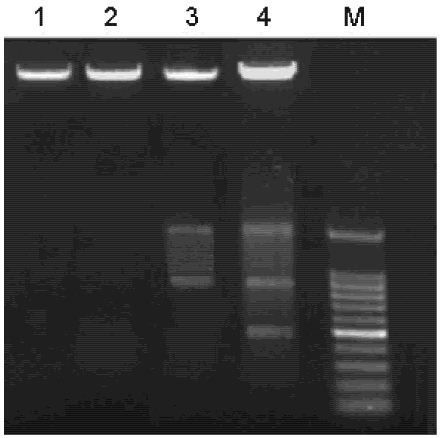
DNA degradation in A549 cells treated with Syngnathusin. The total DNA was extracted and analyzed by electrophoresis on 1.0% agarose gel. 1: control; 2: treated with Syngnathusin (0.25 mg/mL) for 24 h; 3: treated with Syngnathusin (0.5 mg/mL) for 24 h; 4: treated with Syngnathusin (1.0 mg/mL) for 24 h; M: 100 bp DNA Marker.

To further understand the molecular events underlining the Syngnathusin on tumor cells, DNA ladder assay was performed to study the apoptosis induced by Syngnathusin. Although the fragmentation was not observed in the cells treated with 0.25 mg/mL, the agarose electrophoresis of DNA from the cells treated with 0.5 and 1.0 mg/mL Syngnathusin did display DNA ladders, demonstrating that the obvious apoptosis occurred (Figure 9). 

##### 2.3.2.3. Effect on Tumor Cells Morphology

**Figure 10:**
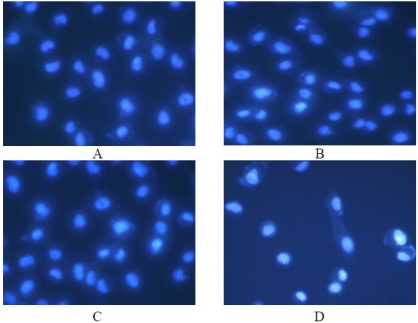
Effect of protein Syngnathusin on A549 cells morphology, detected by fluorescence microscope with Hoechst 33342 staining. (**A**) blank; (**B**) 0.04 mg/mL; (**C**) 0.2 mg/mL; (**D**) 1.0 mg/mL.

Under the fluorescent microscope, the morphology changes of A549 cells were observed. The untreated cells were stained evenly blue fluorescence which showed the chromatin equably distributed in nucleolus and displayed normal, healthy shape demonstrated by the clear skeletons (Figure 10A). However, the morphology of the cells started to change after treatment with Syngnathusin. The cells incubation with the different concentrations of Syngnathusin for 24 h displayed chromatin congregated and nucleolus pyknosis, which emitting bright fluorescence, the early phenomena of apoptosis; the cells detached from the substratum, became round in shape. Membrane bulge and detachment from cytoplasmic inclusion were also observed in the cells treated with Syngnathusin (Figure 10B–D). 

#### 2.3.3. Cooperation with Chemotherapeutics

To further evaluate the antitumor activity of Syngnathusin, its synergetic effects with commonly used chemotherapeutics were studied. Although Syngnathusin was not observed to strengthen the proliferation inhibition of MTX toward LH1210 cells, the results did show that Syngnathusin could enhance the antitumor activity of MTX toward CCRF-CEM cells. The synergism index (SI) was 2.0, indicating there was an obvious synergetic effect between MTX and Syngnathusin (Table 4).

**Table 4:** The synergistic inhibition of combinationwith protein Syngnathusin and commonly use chemotherapeutics (*n* = 3).

Synergism drug	SI	
	CCRF-CEM	L1210
MTX	2.0 ± 0.2	0.9 ± 0.1
5-Fu	0.8 ± 0.1	0.8 ± 0.1

Syngnathusin concentration: 0.20 mg/mL; MTX concentration: 0.13, 0.25, 0.50 μg/mL; 5-Fu concentration: 2.5, 5.0, 10 μg/mL. SI ≥ 1.5, synergetic effect; SI ≤ 0.5, antagonistic effect; 0.5 < RI < 1.5, additive effect.

**Figure 11:**
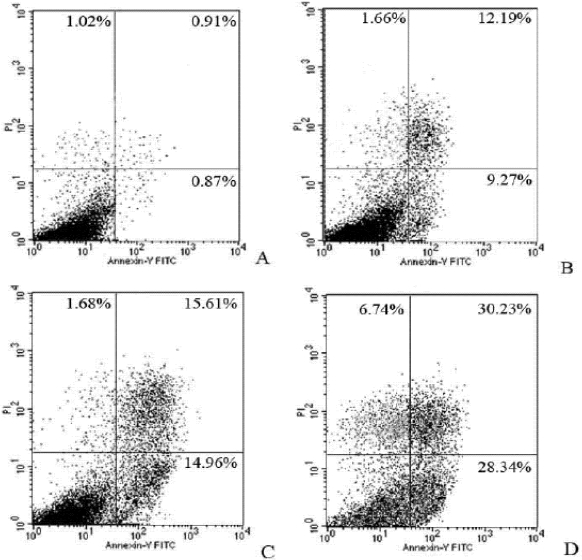
Combination effect of protein Syngnathusin and MTX on CCRF-CEM cells. Treated for 24 h. (**A**) control; (**B**) 0.20 mg/mL Syngnathusin; (**C**) 0.25 μg/mL MTX; (**D**) 0.20 mg/mL Syngnathusin *+* 0.25 μg/mL MTX. UL: dead; LL: alive; UR: late apoptosis; LR: early apoptosis.

The synergetic effect with Syngnathusin and MTX was further evaluated by flow cytometer with Annexin V-PI double staining. The results suggested that both early apoptotic cells and non-viable apoptotic cells were increased in wider band, indicating Syngnathusin enhanced the CCRF-CEM cells apoptosis induced by MTX (Figure 11).

## 3. Experimental Section

### 3.1. Materials

*Syngnathus acus* L. was collected from Haikou, Hainan Province (China) in July 2008, and identified by one of authors Xiaobo Li. A voucher specimen (SJTU 20080719) was deposited in the School of Pharmacy, Shanghai Jiao Tong University.

DEAE Sepharose Fast Flow and Superdex-75 were obtained from Amersham, USA. RNase A and TX0112 Dialysis bags (molecular weight cut-off 14 kDa) were provided by Sangon bioengineering Ltd., Inc., China. MTT, Tris, SDS, Hoechst 33342, acrylamide and PI were purchased from Sigma, USA. Trysin-EDTA was provided by Biochrom AG, Germany. Coomassie brilliant blue G-250 and bovine serum albumin were obtained from Sino-American Biotechnology Co., USA. Molecular weight markers were obtained from Fermentas, Lithuania. 100 bp DNA marker was provided by MBI, USA. MTX, CTX, and 5-Fu were provided by Shanghai Hualian Pharmaceuticals Co., Ltd, China. Universal Genomic DNA extraction kit was provided by Takara Bio. Inc., Japan. All other chemicals and reagents used were of analytical grade.

### 3.2. Cell Lines and Animals

Four tumor cell lines (A549, CCRF-CEM, LoVo, L1210), and six human non-tumor cell lines (HCF, HE-21, HFL-1, HL-7702, PBMC, RPTEC) were provided by Shanghai Institutes for Biological Sciences, Chinese Academy of Sciences. 

Male ICR mice (6 weeks old) with an average body mass of 20.0 ± 1.0 g at the time of study initiation, provided by Shanghai Laboratory Animal Center of Chinese Academy of Sciences (Certificated No: SCXK (Hu) 2007-0018). The animals were housed ten per cage at 22 ± 1 °C and 50 ± 10% relative humidity, and subjected to a 12 h light/12 h dark cycle. They were acclimatized for 5 days before use and provided food and water *ad libitum*. The use of animals in this project was approved and in compliance with the regulations of the Institutional Animal Care and Use Committee of the School of Pharmacy, Shanghai Jiao Tong University, Shanghai, China. 

### 3.3. Bioassay-Guided Isolation of Antitumor Protein

All purification steps were carried out at 4 °C. The piece (100 g) of the whole body of *S. acus* was washed with tap water two times and then extracted with 800 mL distilled water for 12 h under continuous stirring. After centrifugation (3000 g, 30 min), the supernatant was collected and subsequently fractionated by salting out with ammonium sulfate, up to 80% saturation in 30 min. The protein precipitate was collected by centrifugation (4800 g, 30 min), dissolved in 30 mL of distilled water and dialyzed against 3 L distilled water for 6 h three times to completely remove residual ammonium sulfate. The dialyzed solution was freeze-dried to give the crude protein of *S. acus* (CPAS).

CPAS was loaded onto a DEAE Sepharose Fast Flow column which was preequilibrated with 20 mM Tris-HCl buffer (pH 8.0), and then eluted with increasing concentration of NaCl prepared in 20 mM Tris-HCl buffer. The fractions eluted were collected, dialyzed against distilled water and freeze-dried for the proliferation inhibition assay. The fraction with the optimal activity was subsequently injected into a Superdex-75 column and eluted with 0.2 M NaCl prepared in 20 mM Tris-HCl buffer. Each peak fraction was collected, dialyzed against distilled water and freeze-dried for the subsequent proliferation inhibition assay.

Proliferation inhibition *in vitro* was evaluated by MTT method [20], and expressed as IC_50_, *i.e.*, the sample concentration able to inhibit cell growth by 50% compared with the untreated control. All experiments were carried out in triplicate, and data in the form of mean ± SD were presented.

### 3.4. Characterization of Antitumor Protein

#### 3.4.1. Molecular Weight Determination

The molecular weight of protein was determined by SDS-PAGE and MALDI-TOF-MS.

*SDS-PAGE*: SDS-PAGE was performed with a mini-gel apparatus (VE-180 vertical electrophoresis bath, Tanon, China). The samples were analyzed according to the method in Laemmli [21], using an acrylamide concentration of 5% for the stacking gel and 12% for the running gel. Protein bands were detected by GIS 2010 Gel Imaging System (Tanon, China) and molecular weight was calculated according to the standard curve.

*MALDI-TOF-MS*: Protein sample was mixed with matrix solution (sinapinic acid in 50% acetonitrile and 0.1% trifluoroacetic acid) and the mixture was spotted on the MALDI target plate. The spectrum was acquired in positive ion mode at 20 kV in linear mode on a MALDI-4800-TOF-TOF-MS (ABI Inc.).

#### 3.4.2. Isoelectric Point Determination

Isoelectric point of protein sample was determined by immobilized pH gradient (IPG) isoelectric focusing-polyacrylamide gel electrophoresis (IEF-PAGE), which was carried out on PROTEAN IEF Cell (BIO-RAD) according to the manufacturer’s protocol. The gel was made with ampholine at pH range from 4.5 to 9.6 and stained with coomassie brilliant blue R250 [22].

#### 3.4.3. Carbohydrate Concentration Assay

The total neutral sugar content of the purified protein was determined by the modified version of the colorimetric phenol-sulfuric acid method using glucose as standard. The absorbance at 490 nm was used to determine the amount of carbohydrate in the sample [23].

#### 3.4.4. Analysis of Amino Acid Composition

The composition of amino acids was analyzed by an L-8900 amino acid automatic analyzer (Hitachi, Japan). Sample was applied into hydrolysis tube and hydrolyzed with 6 M HCl at 110 °C for 18 h. The hydrolysate was dried, dissolved in 0.02 N HCl, and centrifuged at 10,000 rpm for 15 min. The amino acid compositions were then obtained by automatic analysis algorithm of the amino acid automatic analyzer. 

#### 3.4.5. Analysis of the *N*-Terminal Amino Acid Sequence

The purified protein was electroblotted to PVDF membrane after SDS-PAGE. The transferred visualized bands in PVDF membrane were cut and applied to a protein sequencer PROCISE 491 (Applied Biosystems, USA). The amino acid sequence was analyzed by Edman degradation, with chemicals and the program supplied by the manufacturer. Homology search of N-terminal sequence was performed using BLAST software in NCBI. 

### 3.5. Antitumor Evaluation

#### 3.5.1. Evaluation of Antitumor Activity *in Vivo*

*Solid tumors*: ICR mice were inoculated hypodermically on the left flank with 1 × 10^7^ cells of S180. Samples and CTX (positive control) were administered intraperitioneally (*i.p.*) once a day for 7 days starting from 24 h after tumor implantation, and mean tumor weight (MTW) of each group was examined on 11th day. Antitumor activity was evaluated in terms of the percentage of reduced tumor weight (growth inhibition %) over the control group. Growth inhibition (%) = (1 − MTW of treated group/MTW of control group) × 100 [24]. 

*Ascitic tumors*: ICR mice were inoculated (*i.p.*) with 1 × 10^6^ cells of H22 and randomly divided into 5 groups of mice. Samples, NS and 5-Fu were administered (*i.p.*) once a day for 7 days starting from 24 h after tumor implantation. The mean survival time (MST) of each group was examined on the 9th day. Antitumor activity was evaluated in terms of life prolongation rate calculated by the following: lifeprolongation rate (%) = (MST of treated group/MST of control group − 1) × 100 [24].

#### 3.5.2. Induction of Apoptosis in Tumor Cells

##### 3.5.2.1. Cell Cycle Analysis

A549 cells were seeded at 1 × 10^6^/mL in six-well plates and treated with sample. After incubation for 24 h, the cells were collected and washed with PBS for three times and then were resuspended in 5 μg/mL PI (1 mL) containing 100 U/mL RNase A. The DNAs of cells were stained by PI for 30 min, while the RNAs were removed by digestion with RNase A at 4 °C for 30 min. The DNA contents of the cells were determined at an excitation wavelength of 488 nm by FACScan (BD Biosciences, CA, USA).

##### 3.5.2.2. Comet Assay

A549 cells were seeded at 5 × 10^6^/mL in six-well plates and incubated for 24 h. After treated with sample for 24 h, the cells were collected and total genomic DNA was extracted according to the protocol described in Universal Genomic DNA extraction kit (Takara, Japan). The DNA samples were resolved on 1.0% agarose gel, electrophoresis, stained with 0.5 μg/mL of ethidium bromide and photographed by GIS 2010 Gel Imaging System (Tanon, China).

##### 3.5.2.3. Cell Morphological Assessment

A549 cells were seeded at 1 × 10^5^/mL in 24-well plates and treated with different concentration samples (40, 200 and 1000 μg/mL, respectively) for 24 h. After the treatment, all floating and attached cells were harvested with 0.02% (w/v) EDTA and 0.25% (w/v) trypsase, fixed with 4% (w/v) paraformaldehyde for 20 min and permeabilized by 0.2% Triton X-100 for 10 min. When washed with PBS buffer, cells were stained by fluorochrome dye Hoechst 33342 for 15 min in dark and observed under an inverted fluorescence microscope (Olympus IX51, Tokyo, Japan) with a peak excitation wavelength of 340 nm.

#### 3.5.3. Cooperativity with Chemotherapeutics

The inhibitions of only chemotherapeutics and combination of chemotherapeutics and protein sample (0.20 mg/mL) were determined by MTT, respectively. SI was calculated by the following formula: SI = I_c+p_/(I_c_ + I_p_ − I_c_ × I_p_), I_c_ and I_p_ mean inhition of chemotherapeutics and protien, I_c+p_ means inhibition of combination of chemotherapeutics and protein [25]. To further confirm the synergic action between chemotherapeutics and protein, their induction apoptosis on Lovo cells was further evaluate by flow cytometer with Annexin V-PI double staining.

### 3.6. Statistical Analysis

The results were expressed as mean ± SD. Differences between groups were evaluated by one-way analysis of variance (ANOVA) followed by Dunnett’s test with the aid of SPSS 16.0 software package [26]. Statistical significance is expressed as * *p* <0.05, ** *p* < 0.01.

## 4. Conclusions

A novel protein Syngnathusin was successfully purified from *Syngnathus acus* by bioactive guideline isolation. It showed strong and selective antitumor activities *in vivo* and *in vitro*. In addition, it could combine the antitumor activities with MTX. Syngnathusin is a promising protein for the development of a new antitumor agent.

## References

[1] Sun H.Y., Hou X.N., Wang C.M. (2001). Recent advances in bioactive proteins and peptides from traditional Chinese medicine. J. Chin. Med. Mater..

[2] Yu R.M., Yan C.Y., Qu H.Y., Yao X.S. (2004). Progress and perspective of studies on the bioactive polypeptide from marine products. Mar. Sci. Bull..

[3] Zhang B., Wu W.T. (2004). Advances on studies of marine active antitumor proteins and peptides. Chin. J. Mar. Drugs.

[4] He L.H., Huang J.H., Sheng S.D., Sun S.Y. (2005). Anti-tumor substances of shark and their action mechanisms. Mar. Sci..

[5] Shen X.R., Jia F.X., Zhou J.Y., Chu Z.Y., Wang L., Jiang D.W. (2001). Anti-tumor effect of the preparation extracted from sea fish *Manta birostris*. Chin. J. Mar. Drugs.

[6] Song L.Y., Ren S.F., Yu R.M., Yan C.Y., Li T.F., Zhao Y. (2008). Purification, characterization and *in vitro* anti-tumor activity of proteins from *Arca subcrenata* Lischke. Mar. Drugs.

[7] Li L.J., Wen Y.M., Wang C.M., Chen J. (2002). Experimential studies on protamine of the vascularization inhibition and induction tumor cells apoptosis. Chin. Oncol..

[8] Rajanbabu V., Chen J.Y. (2011). Applications of antimicrobial peptides from fish and perspectives for the future. Peptides.

[9] China Pharmacopoeia Committee (2010). Chinese Pharmacopoeia (I).

[10] Wu C.W., Chen X.E., Wang Z.Z., Wang W.H. (1996). The analysis of nutritional ingredients in *Syngnathus acus*. J. Zhejiang Coll. Fish..

[11] Zhang Z.H., Xu G.J., Xu L.S., Wang Q., Namba T., Kadota S. (1998). Chemical study on *Syngnathus acus* L.. Chin. Trad. Herb. Drugs.

[12] Li S.M., Wu X.D., Zeng S., Luan L.J., Shao Q. (2001). Study on anticancer activity of *Syngnathus*
*in vitro*. Chin. J. Chin. Mater. Med..

[13] Zhang Z.H., Xu G.J., Xu L.S., Wang Q. (1995). Hormonic effects of the ethanol extracts of *Syngnathus*. J. Chin. Med. Mater..

[14] Gao H., Pan X.Q., Yang Y.S. (1982). The estrogen-like effects of *Syngnathus*. Chin. J. Mar. Drugs.

[15] Hu J.Y., Li B.F. (2002). Research on anti-fatigue effect of *Syngnathus acus* L.. Chin. J. Mar. Drugs.

[16] Feng W.B., Tang X.L., Li G.Q., Hai F., Yan J., Xu J. (2010). Comparison study on the compositions and pharmacological activities for *Solenognathus hardwickii* and *Syngnathus acus*. Chin. J. Mar. Drugs.

[17] Li C.X., Bian H.R., Zou G.L., Zhu X.M., Ju X.S. (2001). Studies on anti-cancer effect of Hailong extracts from *Syngnathus acus* Linnaeus. Wuhan Univ. J. (Nat. Sci. Ed.).

[18] Li C.X., Wu W.Q. (2009). Study on effect of *Syngnathus acus* Linnaeus extracts on mice transplanted tumor. J. Anhui Agric. Sci..

[19] Li C.X., Yuan Q., Zhu X.M., Yin S.Q. (2001). The killing effect of *S. acus* Linnaeus exact on Hela. J. Tangshan Teach. Coll..

[20] Mosmann T. (1983). Rapid colorimetric assay for cellular growth and survival: application to proliferation and cytotoxicity assays. J. Immunol. Meth..

[21] Laemmli U.K. (1970). Cleavage of structural proteins during the assembly of the head of bacteriophage T4. Nature.

[22] Righetti P.G. (1990). Immobilized pH Gradients: Theory and Methodology.

[23] White C.A., Kennedy J.F., Chaplin M.F., Kennedy J.F. (1986). Oligosaccharides. Carbohydrate Analysis, a Practical Approach.

[24] Kim J.H., Lee S.J., Han Y.B., Moon J.J., Kim J.B. (1994). Isolation of isoguanosine from *Croton tiglium* and its antitumor activity. Arch. Pharm. Res..

[25] Dai T.J. (1998). Quantitative analysis of drug combination. Chin. Pharm. Bull..

[26] (2007). SPSS for windows.

